# Canada’s Enhanced Dementia Surveillance Initiative: Improving national dementia measures to inform public health actions

**DOI:** 10.1002/alz.089731

**Published:** 2025-01-09

**Authors:** Larry C Shaver, Danielle Marion, Catherine Pelletier

**Affiliations:** ^1^ Public Health Agency of Canada, Ottawa, ON Canada

## Abstract

**Background:**

The Enhanced Dementia Surveillance Initiative (EDSI), led by the Public Health Agency of Canada (PHAC), supports the implementation of Canada’s first national dementia strategy. To improve the national monitoring of dementia and its health impacts, the EDSI projects focused on priority data gaps: dementia by cause, progression stages and impacts; socio‐demographic characteristics, risk and protective factors; and caregivers.

**Method:**

PHAC collaborated on 15 projects with multiple stakeholders (universities/research institutions, health organizations, and federal/provincial government departments). Each employed novel methods and data sources in the Canadian public health surveillance context. Existing and linked health administrative data, electronic medical records (EMR), various primary data collections, and longitudinal cohorts were leveraged to apply new approaches addressing the listed data gaps.

**Result:**

Projects enabled various surveillance advancements in dementia health measures; examples are presented under four categories.

1. By leveraging existing databases with new linkages, algorithm validation and data visualization techniques, projects were able to better identify and characterize (clinical and sociodemographic characteristics) people living with dementia and their caregivers.

2. By enhancing linked health administrative data for national surveillance, new methods to report on dementia in long term care and dementia comorbidities were developed, and health care costs across dementia progression stages were calculated.

3. By developing new conceptual and data models, it was possible to enhance the understanding of the dementia national data landscape. The impact of selected factors on dementia risk in the population was also estimated, and dementia patients’ trajectories across care settings were described.

4. By applying a health equity lens, progress was made towards an improved monitoring of dementia in diverse populations, such as Indigenous communities and individuals experiencing homelessness.

**Conclusion:**

Through a knowledge synthesis of the results and findings from the EDSI projects, PHAC aims to identify and integrate best practices for national dementia surveillance. The expected long‐term outcome of this initiative is to contribute to more robust evidence on dementia in Canada to help inform related public health actions.

